# Food Preferences and Acceptability of Molded Pureed Foods in Community-Dwelling Older Adults in Singapore

**DOI:** 10.3390/nu18203413

**Published:** 2026-10-18

**Authors:** Verena Ming Hui Tan, Gary Tung Lin Chiah, Ives Yubin Lim, Wai Yee Mah, Peng Peng Lim, Kenny Ban Leong Tan

**Affiliations:** 1 Dietetics and Nutrition, Health and Social Sciences, Singapore Institute of Technology, Singapore 828608, Singapore; gary.chiah@singaporetech.edu.sg; 2 Precision Health, Bioinformatics Institute, Agency for Science, Technology and Research (A*STAR), 30 Biopolis Street, Singapore 138671, Singapore; ives_lim@a-star.edu.sg; 3 St. Luke’s ElderCare Ltd., 461 Clementi Road, Singapore 599491, Singapore; waiyeemah@slec.org.sg (W.Y.M.); pengpenglim@slec.org.sg (P.P.L.); kennytan@slec.org.sg (K.B.L.T.)

**Keywords:** texture-modified diet, dysphagia, older adult, food preference, sensory evaluation

## Abstract

Background/Objectives: Texture-modified diets (TMDs) are prescribed for older adults with chewing or swallowing difficulty, but conventional pureeing dilutes nutrient density and yields visually indistinguishable meals that discourage intake. This study aimed to compare food preferences and sensory priorities by self-reported chewing/swallowing difficulty status and to conduct a preliminary evaluation of the sensory acceptability of molded pureed meal prototypes. Methods: An interviewer-administered survey of 100 community-dwelling older adults in Singapore characterized chewing and swallowing symptoms, TMD status, sensory-attribute importance, meal satisfaction, and food and snack preferences. In a separate exploratory pilot, 16 older adults without self-reported swallowing difficulty evaluated four enzymatically modified, molded pureed prototypes on a 7-point hedonic scale. Results: Fifty-six participants (56.0%) reported chewing and/or swallowing difficulty. No statistically significant differences were observed between groups in age, BMI, sex, marital status, sensory-attribute importance, meal satisfaction, or food/snack category preferences (all *p* ≥ 0.05). Among those with difficulty, only 19 (33.9%) had been recommended a TMD and 15 (26.8%) were currently consuming one, while 37 (66.1%) had neither. All four molded pureed prototypes had mean hedonic scores for individual attributes ranging from 4.7 to 6.6 on the seven-point scale. Overall acceptability was high (8.1 ± 1.4 of 10) and most participants were willing to prepare (93.8%), purchase (81.3%), or incorporate (68.8%) the meals. Conclusions: Chewing and swallowing difficulty was common and was not associated with differences in food preferences. Texture-modified meals may be developed from familiardishes, informed by earlier community screening.

## 1. Introduction

Aging alters the anatomy and physiology of eating and swallowing. Older adults have reduced tongue pressure, reduced salivary flow, and impaired dentition and require more chewing cycles and longer oral transit times than younger adults [1]. These changes occur alongside anorexia of aging and altered taste perception [2]. Oral frailty, which combines several of these changes, is associated with poorer quality of life and reduced survival among long-term care residents [3,4]. Impaired swallowing also raises the risk of choking and aspiration. Affected individuals may become fearful of eating and derive less enjoyment from food, which further reduces intake and contributes to malnutrition, sarcopenia, and frailty [5,6].

Estimates of dysphagia prevalence in community-dwelling older adults vary considerably by screening method. A recent systematic review and meta-analysis reported a pooled prevalence of 12.1% using water-swallow screening and 30.5% using standardized assessments. Among nursing home residents, the prevalence increased to 53.6–58.7% [7]. Population-based cohort data corroborate that self-reported swallowing difficulty is common and more frequent among those with poorer self-rated health and lower physical activity [8]. In Singapore, an estimated 58,000 to 174,000 community-dwelling older adults live with dysphagia [9]. Malnutrition and dysphagia frequency co-occur, yet nutritional assessment in this group is not standardized [10].

Texture-modified diets (TMDs) consist of foods processed to textures that are easier to chew and swallow. They are prescribed following a dysphagia assessment, usually by a speech–language therapist, who determines the appropriate level within the International Dysphagia Diet Standardization Initiative (IDDSI) framework [11]. The purpose of a TMD is to maintain oral nutrition while reducing the risk of choking and aspiration. A meta-analysis of randomized controlled trials found that diet modification improves dietary nutrient intake and reduces aspiration in adults with dysphagia [12]. In community-dwelling older adults, provision of texture-modified food has been shown to reduce chewing and swallowing risk [13].

Despite these benefits, the processes used to achieve the IDDSI levels introduce well-documented drawbacks. First, achieving the lower IDDSI levels, particularly Level 4 (pureed), requires added liquid, which reduces energy and protein density. A recent hospital comparison found that an enriched dysphagia diet delivered approximately twice the daily energy and protein of a conventional homogenized diet [14]. Second, adults consuming TMDs have poorer nutritional status and lower mealtime satisfaction than those on regular diets [15], and the texture level consumed is associated with malnutrition and sarcopenia [6]. Third, pureeing homogenizes separate meal components into a single indistinguishable form. Consumers report dissatisfaction with the appearance, taste, and texture of these meals, and reduced appeal is itself a determinant of intake in older adults [16].

Several approaches have been used to improve the appeal of TMDs, including gelation, enzymatic modification, and extrusion-based three-dimensional printing [17,18]. Enzyme gellants are of practical interest because they set thermally and require no specialized equipment, so meals can be prepared in an institutional or a domestic kitchen. In a Hong Kong study, pureed meals were modified with a commercial enzyme gellant and cast in molds resembling the source ingredients; residents of aged care facilities accepted the modified purees, and swallowing safety was maintained [19]. Another study reported that a texture-modified, enriched and reshaped diet increased energy and protein intake among German nursing home residents with chewing or swallowing problems [20]. Reshaped meals for patients with dysphagia have also been shown to be acceptable to older adults without swallowing difficulty, which matters when meals are shared at a common table [21]. Neither of the reshaping studies adapted the dishes to Southeast Asian cuisine, and Singapore’s multi-ethnic food culture spans Chinese, Malay, and Indian cuisines that differ in preparation and presentation.

Dysphagia remains an underdiagnosed geriatric syndrome outside institutional settings. Systematic community screening has identified more affected individuals than routine referrals [22]. The prevalence of symptoms relative to the proportion who received a formal diagnosis in the community has not been quantified, nor is it known whether the two groups differ in what they want to eat or whether texture-modified meals developed from familiar dishes are acceptable to older adults who have not yet developed a swallowing difficulty. Prior studies of food preference in this population have generally stratified participants by prescribed diet texture rather than by reported symptoms. This is because diet prescription reflects a clinical diagnosis rather than underlying need; hence, this approach cannot establish whether food preferences actually differ between those with and without chewing or swallowing difficulty. Self-reported difficulty does not necessarily mean a prescribed TMD is required. Individuals with mild or intermittent difficulty may adapt their diet informally by chewing for longer or avoiding harder foods without ever being assessed [23]. Such difficulty is not, however, expected to resolve spontaneously with advancing age. Longitudinal cohort studies have found that self-reported eating difficulty and oral frailty more broadly predict progression to physical frailty over subsequent years [24,25].

The aims of the study are (1) to characterize food preferences, sensory priorities, and meal satisfaction among community-dwelling older adults and (2) to evaluate the sensory acceptability of enzymatically modified, molded pureed meal prototypes based on familiar food items.

## 2. Materials and Methods

This study comprised two components conducted among community-dwelling older adults in Singapore. First, an interviewer-administered survey was conducted among older adults attending senior day-care centers. Second, a sensory evaluation pilot of molded pureed meal prototypes was conducted among older adults attending active aging centers. The two components recruited separate, non-overlapping samples from different settings; no participant took part in both. Both components were conducted in accordance with the Declaration of Helsinki. The survey was approved by the Institutional Review Board of the Singapore Institute of Technology (RECAS-0497, approved 12 February 2025), and the sensory evaluation was approved by the same board (RECAS-0746, approved 25 January 2026). Written informed consent was obtained from all participants.

### 2.1. Interviewer-Administered Survey

One hundred community-dwelling adults aged 60 years and above were recruited by convenience sampling from 14 senior day-care centers in Singapore between March and October 2025. Participants were eligible if they were able to give informed consent and complete an interviewer-administered survey in English or Mandarin. Participants were excluded if they had been diagnosed with Alzheimer’s disease, dementia or other conditions that result in cognitive impairment or were unable to communicate in English or Mandarin. No a priori sample size calculation was performed. The target of 100 participants was set by available recruitment resources and is comparable to that of a previous survey of food preferences among community-dwelling older adults [26].

The survey was administered face to face by a trained research dietitian and took approximately 20 min to complete. The Mandarin version was translated from the English version by a bilingual research dietitian and back-translated by the same dietitian. The back-translated version was checked against the original English version by the corresponding author. Demographic items included age, sex, ethnicity and marital status. Clinical information included denture use, current diet texture, expressed as an IDDSI level, and whether a TMD had ever been recommended by a healthcare professional. Chewing difficulty and swallowing difficulty were each assessed with a single self-reported yes/no item. Participants answering affirmatively to either item were classified as having chewing and/or swallowing difficulty.

Meal-related attitudes were assessed with eleven items. Four items rated the importance of taste, smell, appearance, and resemblance to previously enjoyed foods on a five-point scale from 1 (not at all important) to 5 (very important). Four items rated satisfaction with the appearance, taste, smell, and texture of meals currently served at the participating centers from very dissatisfied to very satisfied. Three items rated agreement that greater variety, improved seasoning and flavor, and better presentation and plating would make meals more enjoyable from strongly disagree to strongly agree. Usual main-meal portion size was recorded separately using a five-point pictorial scale ranging from less than one-quarter to a full share.

Food and snack preferences were elicited by free recall. Participants named up to five favorite foods and up to five favorite snacks. Responses were coded into standardized categories by two researchers working independently; disagreements were resolved by discussion until consensus was reached.

### 2.2. Preparation of Molded Pureed Food Items

Four food items were selected: teriyaki chicken with porridge, stir-fried long cabbage, prawn dumpling with thickened soup and marble cake. Selection was guided by the food categories most frequently recalled in the survey component and covered the main components of a local meal with a dessert item. All four were purchased ready-prepared from commercial outlets.

Purees were prepared using an enzyme gellant (MAKANsoft, DIGNIFIED programme, Singapore Institute of Technology, Singapore). MAKANsoft is an enzyme-containing gelling blend that reduces the stickiness of pureed food and sets it into a gel upon cooling. Each food item was weighed and placed in a food blender (Robot-Coupe, Robot Cook, Vincennes, France), and water was added based on the food-to-liquid ratio indicated in Table 1. The amount of enzyme gellant was added as percentage weight of the total weight of the pureed food and liquid. Table 1 shows the preparation parameters for the four molded pureed prototypes.

**Figure 1:**
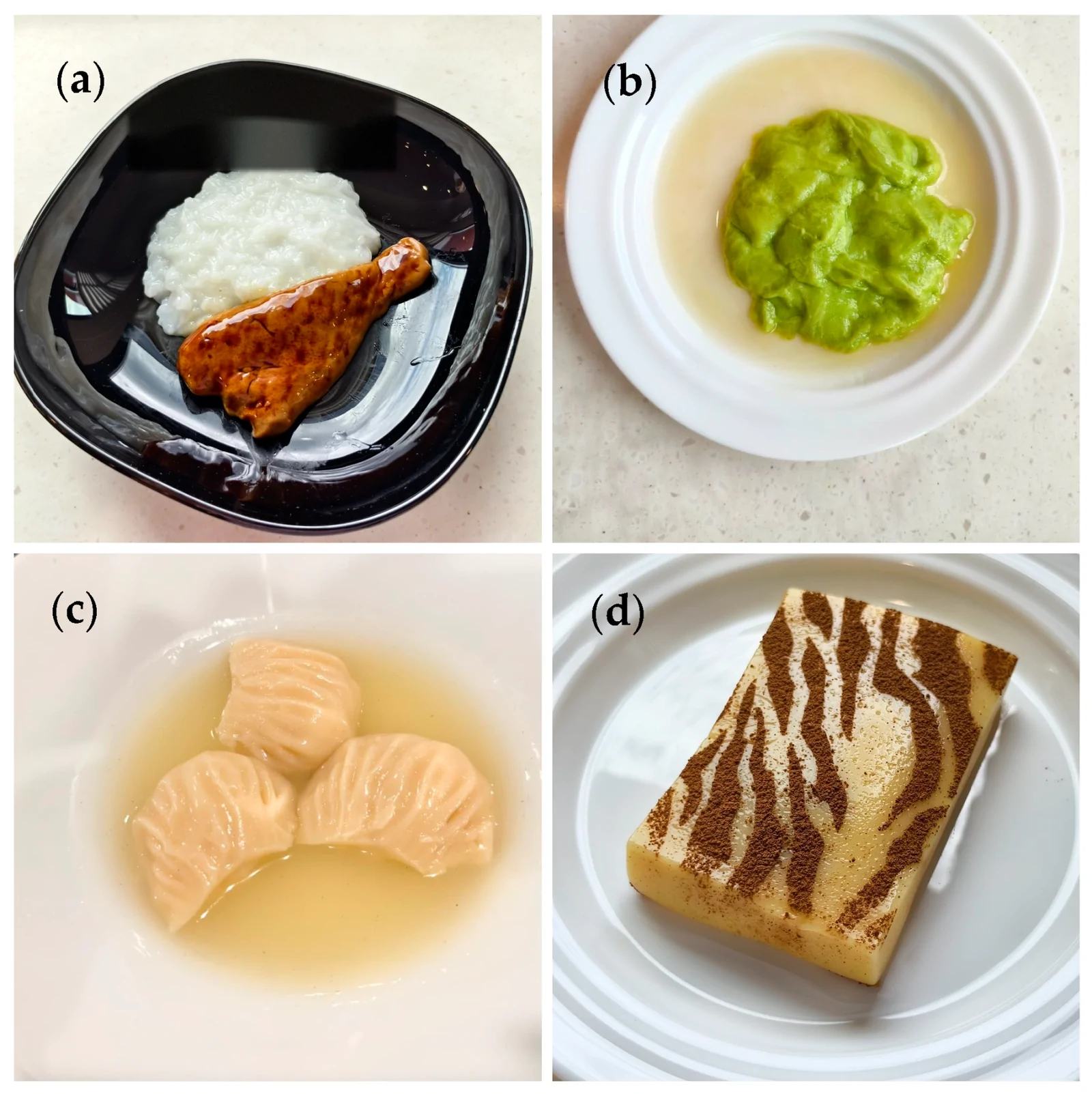
Molded pureed prototypes: (**a**) teriyaki chicken with porridge; (**b**) stir-fried long cabbage; (**c**) prawn dumpling with thickened soup; (**d**) marble cake.

The combined pureed food and enzyme gellant was blended for 3–4 min. The blended mixture was transferred to a shallow pan and heated on the lowest setting with stirring. Once bubbling was observed, the pan was covered, agitated, and heated for a further 5 min until the mixture was fully liquefied, ensuring it exceeded the 70 °C required to activate the gelling agents and enzyme. The liquid mixture was promptly poured into food-grade silicone molds that resembled the shapes of the original food item (Figure 1) and left to set for 20 min at ambient temperature. Once cooled, the prototypes were unmolded onto serving plates and served warm. Each prototype was assessed against the IDDSI framework and validated to meet the IDDSI level 4 (pureed) using the spoon tilt test and fork drip test [11].

### 2.3. Sensory Evaluation of Molded Pureed Food Items

In an exploratory pilot, sixteen community-dwelling older adults were recruited from one active aging center. Participants were eligible if they were members of St. Luke’s ElderCare Active Aging Centre, aged 60 years and above and were able to come to the SIT for the sensory session. Participants were excluded if they had any food allergies, intolerances, restrictions or problems with tasting and smelling food. Recruited participants were untrained consumers and had no prior exposure to the prototypes.

The sensory evaluation session was conducted in the dietetics lab at the Singapore Institute of Technology. Participants were seated at separate tables and evaluated the samples independently. The four prototypes were presented monadically in a fixed order that was the same for all participants. Each sample was served at approximately 40 °C. Participants were told the name of the dish each prototype represented and were shown a photograph of the original food. Plain water was provided as a palate cleanser between samples.

Participants rated each prototype on appearance, texture, taste, and smell using a 7-point hedonic scale anchored at 1 (dislike very much) and 7 (like very much). After evaluating all four prototypes, participants rated the overall acceptability of the molded pureed meal concept on a 0–10 scale and answered three yes/no questions regarding their willingness to incorporate such meals into their regular diet, to purchase them, and to prepare them at home.

### 2.4. Statistical Analysis

The primary grouping variable for the survey was self-reported chewing and/or swallowing difficulty, selected to match the symptomatic population the study was designed to characterize. Current TMD status was analyzed as a descriptive outcome, and comparisons stratified by TMD status were conducted as a secondary analysis. In sensitivity analyses, chewing difficulty and swallowing difficulty were each used separately as the grouping variable.

Non-parametric methods were used for continuous and ordinal outcomes, given the nature of the response scales and distribution of data. Group differences in ordinal Likert-scale responses between individuals with and without swallowing or chewing difficulties were evaluated using asymptotic Wilcoxon rank-sum tests via the coin package. Effect sizes were calculated as the absolute standardized z-statistic divided by the square root of the total sample size (N). The 95% confidence intervals (CIs) for effect sizes were derived via non-parametric bootstrapping with 5000 resamples, using stratified independent replacement within each difficulty group.

For food and snack preferences, associations between group membership and food or snack recall were assessed using Fisher’s exact test. Odds ratios were estimated by conditional maximum likelihood, and their 95% CIs were calculated from the corresponding contingency tables. Each participant contributed multiple recall items. Responses were therefore recoded per participant as presence or absence within each category before testing. Categories with fewer than 10 participants overall, or fewer than three participants in each subgroup, were flagged as sparse and are reported in Supplementary Table S1.

For the sensory evaluation, differences across the four prototypes were tested separately for each attribute using the Friedman test. Where the Friedman test was significant, pairwise comparisons were made using the Wilcoxon signed-rank test.

The Benjamini–Hochberg procedure was applied separately to each predefined family of comparisons to control the false discovery rate (FDR). In the survey, the families were Likert-scale outcomes, food category preferences and snack category preferences. In the sensory evaluation, each family comprised the six pairwise comparisons within an attribute. Significance was set at two-sided α = 0.05 for uncorrected tests and FDR < 0.05 for corrected comparisons. Analyses were performed using R (version 4.2.1).

## 3. Results

### 3.1. Demographics and Clinical Characteristics by Chewing/Swallowing Difficulty Status

The mean age of participants was 79.9 ± 8.9 years; 58% were female and 93% were of Chinese ethnicity. Fifty-six participants (56.0%) reported chewing and/or swallowing difficulty (33 chewing only, 6 swallowing only, 17 both) and 44 (44.0%) reported neither. No statistically significant differences were observed between groups in age, body mass index, sex or marital status (Table 2). Denture use differed significantly between groups and was more common among participants without chewing or swallowing difficulty (86.4% versus 55.4%, *p* = 0.001). Among the 56 participants reporting chewing and/or swallowing difficulty, 15 (26.8%) were currently consuming a texture-modified diet and 19 (33.9%) had, at some point, been recommended one by a healthcare professional. Thirty-seven participants (66.1%) had neither been recommended a texture-modified diet nor were consuming one.

### 3.2. Meal-Related Attitudes and Satisfaction

None of the 11 meal-related attitude items, nor the item regarding usual main-meal portion size, differed significantly between groups (Table 3). Both groups rated taste as the most important sensory attribute and reported comparable, moderate satisfaction with their current meals across appearance, taste, smell and texture.

In a secondary analysis stratifying by current TMD status, importance of taste and agreement that better presentation would improve enjoyment differed significantly between groups after FDR correction (both FDR = 0.031; Table 4).

### 3.3. Food and Snack Preferences

Twelve food categories and eleven snack categories were derived from the recall responses. Chinese noodle dishes were the most frequently recalled food category in both groups, followed by chicken and pork dishes. Savory and sweet snacks were the most frequently recalled snack categories. No food or snack category differed significantly by chewing/swallowing difficulty status (Table 5). Sensitivity analyses using chewing difficulty (*n* = 50) and swallowing difficulty (*n* = 23) as separate grouping variables likewise showed no statistically significant differences in meal-related attitudes or food and snack recall.

### 3.4. Sensory Evaluation of Molded Pureed Meals

The sensory evaluation was an exploratory pilot conducted in a separate, non-overlapping sample recruited from an active aging center, with the objective of assessing the sensory acceptability of the four molded pureed prototypes. Sixteen older adults evaluated the four molded pureed meals. Their mean age was 71.1 ± 5.8 years, 62.5% were female, and all were of Chinese ethnicity. Three (18.7%) participants reported chewing difficulty, and none reported swallowing difficulty (Table 6). Overall acceptability of the molded pureed meal concept, rated once after all four items had been evaluated, was 8.1 ± 1.4 of 10. Fifteen participants (93.8%) indicated willingness to prepare such meals at home, 13 (81.3%) to purchase them, and 11 (68.8%) to incorporate them into their regular diet if the need arose (Table 6).

All four items were rated above the midpoint of the seven-point hedonic scale on every attribute, with mean attribute scores ranging from 4.7 to 6.6 (Table 7). Ratings differed across the four items in appearance (*p* < 0.001), texture (*p* = 0.002), taste (*p* < 0.001) and smell (*p* = 0.028). Appearance showed the clearest separation: the prawn dumpling and marble cake were rated higher than the teriyaki chicken with porridge and stir-fried long cabbage (all FDR ≤ 0.013), with no statistically significant difference within either pair. The marble cake was also rated higher than the other three items for texture (all FDR ≤ 0.021), and higher than the teriyaki chicken with porridge and stir-fried long cabbage for taste (FDR = 0.024 and 0.013, respectively). No pairwise comparison for smell remained significant after adjustment. Pairwise comparisons for all attributes are given in Table 8.

## 4. Discussion

This study examined food preferences, sensory priorities, and meal satisfaction among community-dwelling older adults and, in a separate group, evaluated the sensory acceptability of enzymatically modified, molded pureed prototypes. It appears that chewing and/or swallowing difficulty was common but largely unaccompanied by a reported TMD recommendation. In addition, in an exploratory pilot, the molded pureed prototypes were accepted by older adults who did not currently report such difficulty. Together, these findings suggest that texture-modified foods may be acceptable to individuals beyond those who currently consume or have been recommended such diets while also highlighting a discrepancy between self-reported difficulties and reported dietary management.

Nearly two-thirds (66.1%) of the participants who reported having chewing/swallowing difficulty had neither been recommended a TMD nor were consuming one. This indicates a discrepancy between self-reported difficulty and reported dietary management but it does not establish an unmet clinical need because prior clinical assessment was not determined. Community-dwelling older adults with dysphagia may conceal their swallowing difficulty to preserve self-identity, with poor adherence to a prescribed texture-modified diet [27]. Oropharyngeal dysphagia is also frequently unrecognized in primary care until hospitalization confirms the diagnosis [28]. The present study did not assess whether participants have undergone formal dysphagia assessment or received other forms of dietary or clinical advice, so self-reported difficulties should not be interpreted as equivalent to a clinical diagnosis of dysphagia. However, the relatively large proportion of participants reporting difficulties without currently consuming a texture-modified diet suggests that opportunities for earlier identification and appropriate dietary assessment may exist in community settings. Prospective studies incorporating validated clinical or instrumental assessments would thus be useful to determine whether self-reported difficulty is associated with subsequent clinical deterioration or the need for TMDs.

Denture use was more common among participants who reported no chewing or swallowing difficulty than among those who did. Unreplaced tooth loss is the most frequently identified risk factor for masticatory dysfunction in older adults, and prosthetic rehabilitation improves both masticatory performance and self-reported chewing ability [29]. Chewing difficulty among participants without dentures may reflect tooth loss that has not been prosthetically addressed, though no dental examination was assessed in this study. Identifying chewing and swallowing difficulty earlier would therefore need to be paired with dental assessment, since a proportion of those difficulties may be resolved by prosthetic rehabilitation rather than requiring a modified diet.

Meal-related attitudes, satisfaction, and food and snack categories showed no statistically significant differences by chewing/swallowing difficulty status in the present study. An independent, larger community survey from our group found the same pattern, identifying ethnicity and cuisine as the primary correlates of texture-modified food attitudes (manuscript, submitted). Classifying participants by self-reported difficulty rather than by prescribed TMD level did not reveal a distinct set of food preferences. This suggests that texture-modified options should be built around familiar food items acceptable to older adults across the population and not only to those already consuming a modified diet. A secondary analysis stratifying by current TMD status did find two differences whereby those currently consuming a TMD rated taste as more important and were more likely to agree that better presentation would improve enjoyment than those not on a TMD. This is consistent with a small group who have already transitioned to a modified diet placing importance on the two attributes most compromised by conventional pureeing. The comparison is nonetheless underpowered and unbalanced and should be treated as hypothesis-generating.

The sensory evaluation panel was drawn from a different, largely asymptomatic group. All four prototypes showed positive ratings on every attribute, with appearance showing the largest differences between items in post hoc pairwise comparisons. This is consistent with evidence that reshaping texture-modified food to resemble its original form improves acceptability relative to conventionally homogenized purees [30], including in an Asian long-term care setting [19]. However, the evidence is currently equivocal. Ott et al. reported gains in energy and protein intake from a reshaped, enriched diet without a corresponding gain in stated preference [20]. Conversely, an older study found unshaped purees to be more appealing than molded forms by both younger and older adult panelists [31]. One possibility is that shaped dishes may be perceived as harder to swallow than dishes that are texture-modified without reshaping. Despite contrasting evidence, what is notable here is that acceptability and behavioral intent were evident in a sample of older adults that did not currently require texture modification, suggesting that receptiveness to texture-modified foods may extend beyond individuals who currently require texture modification.

Taken together, no statistically significant differences in food preferences or sensory priorities were observed by symptom status, and molded pureed meals built from familiar items were well received in a small pilot of older adults without swallowing difficulty. These findings support developing texture-modified versions of familiar dishes, rather than restricting product development to those with an established diagnosis. Whether such meals are acceptable to, and improve intake in, people with dysphagia remains to be tested.

The findings also have implications for clinical practice and policy. In community settings, chewing and swallowing difficulty was common and most of those affected had no reported TMD recommendation. One suggestion could be to administer a validated screen such as the Eating Assessment Tool (EAT-10) [32] at intake and annually, referring positive screens to a speech therapist and, where chewing difficulty is reported without dentures, to a dentist.

At the policy level, the WHO Decade of Healthy Ageing (2021–2030) names integrated, primary-level care responsive to older people’s needs and age-friendly community environments among its priority action areas [33]. Singapore’s Project Silver Screen already offers functional screening of vision, hearing and oral health in older adults aged 60 years and above at active aging centers and other community sites [34]. Adding a validated chewing and swallowing item would extend case-finding without new infrastructure, provided positive screens are linked to assessment and intervention [35]. Brief bedside tests such as the 3-ounce water swallow test and the Gugging Swallowing Screen require no specialized equipment and have shown good diagnostic accuracy outside acute hospital settings [36]. Commercially, texture-modified food provision for older adults in Singapore currently rests with a small number of market players offering a limited product range. Other Asian markets facing similar issues have addressed comparable gaps through coordinated government schemes. Japan’s Care Food Association introduced the voluntary Universal Design Food Standard in 2002, and the government-labeled “Smile Care” food scheme followed in 2015, using colour-coded labels to categorize commercially available foods by chewing/swallowing suitability [17]. In Hong Kong, researchers have established quantitative IDDSI-calibrated textural and rheological reference data across 226 food and 93 liquid samples based on common Chinese ingredients and dishes, providing manufacturers with the technical benchmarks needed to reliably develop and classify IDDSI-compliant products from familiar cuisine [37]. A comparable consumer-facing labeling scheme, built on Singapore’s existing IDDSI, could make IDDSI-compliant, texture-modified familiar foods both easier to develop and easier for consumers, caregivers and institutional buyers to identify at the point of purchase, addressing a supply constraint that clinical standardization alone does not resolve.

This study has several strengths. Participants were classified by self-reported chewing/swallowing difficulty rather than by clinical TMD status, which allowed the proportion of symptomatic participants without a reported TMD recommendation to be quantified directly. The survey was interviewer-administered face to face by a trained research dietitian, which supports consistent question delivery and reduces missing or misunderstood responses in an older adult population. Between-group comparisons were reported, multiple comparisons were controlled by the false discovery rate, and the findings were unchanged when chewing and swallowing difficulty were analyzed separately.

Several limitations should be noted. The survey and sensory evaluation were cross-sectional and used independent samples, so no temporal relationship between acceptability and the onset of clinical need can be inferred. Convenience sampling from senior day-care centers and a predominantly Chinese sample limit generalizability to the wider older population and to Malay and Indian older adults. Chewing and/or swallowing difficulty were each assessed using a single, non-validated yes/no question. The survey was, however, interviewer-administered face to face by a trained dietitian, which reduces, though does not eliminate, misclassification arising from misunderstanding the question. Comparisons that were bivariate and exploratory, such as for denture use and current TMD consumption, differed between groups and may also shape meal-related attitudes. In the absence of an a priori sample size calculation, non-significant differences should not be read as equivalence. In the sensory pilot, the absence of a conventional puree comparator and the fixed, named order of presentation mean that the effects of molding cannot be separated from order and expectation effects. Finally, behavioral intent was stated rather than observed, and nutrient density, intake and swallowing safety were not evaluated.

### Future Research Directions

A prospective cohort design following community-dwelling older adults from a pre-symptomatic state through the onset of chewing and/or swallowing difficulty would allow the relationship between acceptability and the emergence of clinical need to be examined directly. Pairing self-reported symptom screening with a validated clinical instrument would separate confirmed clinical need from perceived difficulty that does not meet a threshold for intervention.

## 5. Conclusions

Among community-dwelling older adults in Singapore, chewing and swallowing difficulty was common, mostly present without a reported TMD recommendation, and was not associated with differences in food preferences or sensory priorities. In an exploratory pilot, molded pureed versions of familiar dishes were accepted by older adults without swallowing difficulty. Incorporating a chewing and swallowing screen into existing community health screening and developing texture-modified options from familiar dishes may support timely dietary assessment and intervention.

## Figures and Tables

**Table 1:** Preparation parameters for the four molded pureed prototypes.

Prototype	Category	Food-to-Liquid Ratio	Texturant Added (% of Food Plus Liquid)
Teriyaki chicken with porridge	Meat	1: 1.3	1.2
Stir-fried long cabbage	Vegetables	1: 0.6	1.2
Prawn dumpling with thickened soup	Starchy food	1: 2	2.0
Marble cake	Starchy food	1: 2	2.0

**Table 2:** Demographic and clinical characteristics by chewing/swallowing difficulty status.

Characteristic	No Difficulty (*n* = 44)	Difficulty (*n* = 56)	*p*-Value
Age (years), mean ± SD	79.5 ± 8.4	80.3 ± 9.2	0.536 *
BMI (kg/m^2^), mean ± SD	22.3 ± 5.2	22.7 ± 5.3	0.898 *
Sex, male/female	20/24	22/34	0.548 ^
Ethnicity, Chinese/Malay/Indian	41/2/1	52/2/2	-
Married, *n* (%)	21 (47.7)	21 (37.5)	0.317 ^
Wears dentures, *n* (%)	38 (86.4)	31 (55.4)	0.001 ^
Currently on TMD, *n* (%)	1 (2.3)	15 (26.8)	<0.001 ^
Ever recommended TMD, *n* (%)	5 (11.4)	19 (33.9)	0.010 ^

BMI, body mass index; TMD, texture-modified diet. * Wilcoxon rank sum test. ^ Fisher’s exact test.

**Table 3:** Meal-related attitudes and satisfaction by chewing/swallowing difficulty status.

Category	Item	No Difficulty (*n* = 44)	Difficulty (*n* = 56)	Effect Size	*p*-Value
		Mean ± SD	Mean ± SD	(95% CI)	
Food intake	Food intake	4.02 ± 1.19	3.75 ± 1.25	0.11 (0.01–0.30)	0.253
Sensory importance	Taste	4.02 ± 1.11	4.14 ± 1.17	0.10 (0.00–0.30)	0.320
	Smell	3.80 ± 1.29	3.50 ±1.38	0.10 (0.01–0.30)	0.294
	Appearance	3.50 ± 1.23	3.82 ± 1.31	0.16 (0.01–0.35)	0.102
	Resemblance to original food	3.41 ± 1.39	3.88 ± 1.08	0.16 (0.01–0.34)	0.121
Satisfaction	Appearance	3.30 ± 1.36	3.57 ± 1.16	0.08 (0.00–0.28)	0.408
	Taste	3.39 ± 1.33	3.27 ± 1.21	0.08 (0.00–0.28)	0.443
	Smell	3.41 ± 1.23	3.27 ± 1.12	0.12 (0.01–0.32)	0.236
	Texture	3.61 ± 1.10	3.36 ± 1.10	0.15 (0.01–0.34)	0.143
Improvement	Variety	3.82 ± 0.97	3.95 ± 1.03	0.08 (0.00–0.28)	0.397
	Flavor	3.84 ± 1.33	3.95 ± 1.21	0.02 (0.00–0.23)	0.805
	Presentation	3.75 ± 1.38	4.00 ± 1.24	0.10 (0.00–0.29)	0.322

CI, confidence interval. *p*-values are unadjusted. No comparisons remained significant after FDR correction.

**Table 4:** Meal-related attitudes and satisfaction stratified by current texture-modified diet (TMD) status.

Category	Item	No TMD (*n* = 84)	Current TMD (*n* = 16)	*p*-Value	FDR
		Mean ± SD	Mean ± SD		
Food intake	Food intake	3.85 ± 1.23	4.00 ± 1.26	0.586	0.861
Sensory importance	Taste	3.96 ± 1.19	4.75 ± 0.45	0.005	0.031
	Smell	3.61 ± 1.34	3.75 ± 1.39	0.549	0.861
	Appearance	3.60 ± 1.27	4.12 ± 1.26	0.062	0.187
	Resemblance to original food	3.56 ± 1.27	4.25 ± 0.86	0.048	0.187
Satisfaction	Appearance	3.43 ± 1.23	3.56 ± 1.36	0.511	0.861
	Taste	3.35 ± 1.25	3.20 ± 1.37 (*n* = 15)	0.737	0.884
	Smell	3.35 ± 1.18	3.25 ± 1.13	0.646	0.861
	Texture	3.50 ± 1.06	3.31 ± 1.35	0.831	0.904
Improvement	Variety	3.81 ± 1.04	4.31 ± 0.70	0.079	0.189
	Flavor	3.92 ± 1.22	3.81 ± 1.47	0.904	0.904
	Presentation	3.75 ± 1.36	4.62 ± 0.50	0.005	0.031

Current TMD status was defined as currently consuming a diet at IDDSI Level 4–6 (*n* = 16); all other participants, including those recommended a TMD but consuming a Level 7 diet (*n* = 4), were classified as not currently on a TMD (*n* = 84). Values are mean ± SD on a 5-point scale. *p*-values are from the Wilcoxon rank-sum test; FDR is the Benjamini–Hochberg-adjusted *p*-value within this family of 12 comparisons. One participant did not answer the taste satisfaction item. Importance of taste and agreement that better presentation would improve enjoyment differed significantly between groups after FDR correction (both FDR = 0.031); no other item reached FDR < 0.05.

**Table 5:** Food and snack category recall by chewing/swallowing difficulty status.

Main Meal/Snack	Category	Total Numbers	No Difficulty (*n* = 44)	Difficulty (*n* = 56)	OR (95% CI)	*p*-Value
Main meal	Chinese noodle dishes	80	37	43	0.63 (0.19–1.91)	0.454
	Chicken dishes	62	29	33	0.74 (0.30–1.81)	0.537
	Pork dishes	46	20	26	1.04 (0.44–2.48)	1.000
	Malay dishes	39	15	24	1.44 (0.59–3.58)	0.414
	Seafood dishes	38	19	19	0.68 (0.28–1.65)	0.408
	Other hawker favorites	29	11	18	1.42 (0.54–3.83)	0.509
	Indian dishes	14	5	9	1.49 (0.41–6.14)	0.572
Snacks	Savory snacks ^1^	83	38	45	0.65 (0.18–2.13)	0.592
	Sweet snacks ^2^	80	38	42	0.48 (0.14–1.49)	0.210
	Deep-fried snacks ^3^	48	21	27	1.02 (0.43–2.42)	1.000
	Baos with filling ^4^	30	14	16	0.86 (0.33–2.22)	0.827
	Bread with spreads/fillings ^5^	13	7	6	0.64 (0.16–2.42)	0.553

OR, odds ratio among participants with chewing and/or swallowing difficulty relative to those without, estimated by conditional maximum likelihood with exact 95% confidence interval (CI). *p*-values are from Fisher’s exact test. ^1^ Savory snacks (in decreasing frequency of mentions) include chee cheong fun (35), chwee kueh (33), siew mai (27), har gao (17), png kueh (10), soon kueh (8), yam kueh (7), bak zhang (5), popiah (4). ^2^ Sweet snacks (in decreasing frequency of mentions) include ang ku kueh (19), red bean soup (17), green bean soup (12), cheng tng (12), kueh salat (9), black sesame paste (8), kueh lapis (7), kueh dadar (6), tau suan (6), butter cake (5). ^3^ Deep-fried snacks include curry puff (30), you tiao (13), ham chim peng (5), sardine puff (2). ^4^ Baos with filling include char siew bao (23), meat bao (4), big bao (3), red bean bao (3), lotus paste bao (1). ^5^ Bread with spreads/fillings include kaya butter toast (10), bread with jam (1), bun with ham (1), bread with peanut butter (1).

**Table 6:** Characteristics, overall acceptability, and behavioral intent of sensory evaluation participants (*n* = 16).

Characteristic	*n* (%)/Mean ± SD
Age (years)	71.1 ± 5.8
BMI (kg/m^2^)	23.2 ± 3.1
Gender	
Male	6 (37.5)
Female	10 (62.5)
Chewing difficulty	
Yes	3 (18.7)
No	13 (81.3)
Swallowing difficulty	
Yes	0 (0)
No	16 (100)
Overall acceptability (0 to 10)	8.1 ± 1.4
Incorporate molded pureed meals	
Yes	11 (68.8)
No	5 (31.2)
Purchase intent	
Yes	13 (81.3)
No	3 (18.7)
Preparation of molded pureed meals	
Yes	15 (93.8)
No	1 (6.2)

**Table 7:** Sensory scores for the four molded pureed food items (*n* = 16).

Item	Appearance	Texture	Taste	Smell
	Mean ± SD	Mean ± SD	Mean ± SD	Mean ± SD
Teriyaki chicken with porridge	4.7 ± 1.5	5.6 ± 1.2	5.9 ± 0.8	5.6 ± 1.0
Stir-fried long cabbage	5.1 ± 1.2	5.6 ± 1.2	5.8 ± 0.9	5.6 ± 1.2
Prawn dumpling with thickened soup	6.4 ± 0.9	6.0 ±1.1	6.3 ± 0.9	5.9 ± 0.9
Marble cake	6.6 ± 0.9	6.6 ± 0.8	6.6 ± 0.6	6.3 ± 0.9

**Table 8:** Pairwise post hoc comparisons of sensory scores for the four molded pureed prototypes (*n* = 16).

Comparison	Appearance	Texture	Taste	Smell
Teriyaki chicken with porridge vs. stir-fried long cabbage	0.0 (0.142)	0.0 (0.885)	0.0 (0.518)	−0.5 (0.886)
Teriyaki chicken with porridge vs. prawn dumpling	−2.0 (0.010) *	0.0 (0.200)	−1.0 (0.068)	−0.5 (0.291)
Teriyaki chicken with porridge vs. marble cake	−2.0 (0.010) *	−1.0 (0.021) *	−1.0 (0.024) *	−1.0 (0.063)
Stir-fried long cabbage vs. prawn dumpling	−2.0 (0.013) *	0.0 (0.051)	−1.0 (0.069)	0.0 (0.308)
Stir-fried long cabbage vs. marble cake	−2.0 (0.010) *	−1.0 (0.020) *	−1.0 (0.013) *	−0.5 (0.063)
Prawn dumpling vs. marble cake	0.0 (0.157)	−1.0 (0.020) *	0.0 (0.158)	−0.5 (0.167)
Teriyaki chicken with porridge vs. stir-fried long cabbage	0.0 (0.142)	0.0 (0.885)	0.0 (0.518)	−0.5 (0.886)

Values are the median difference (first item minus second item), with the Benjamini–Hochberg adjusted *p*-value in parentheses. Pairwise comparisons used the Wilcoxon signed-rank test. Negative values indicate that the first item scored lower. * FDR < 0.05.

## Data Availability

The datasets generated and/or analyzed during the current study are available from the corresponding author upon request, subject to approval by the Singapore Institute of Technology Institutional Review Board.

## Supplementary Materials

The following supporting information can be downloaded at https://www.mdpi.com/article/10.3390/nu18203413/s1, Table S1: Complete food and snack category recall counts by chewing/swallowing difficulty status.
