# Supplementary material for: Food Preferences and Acceptability of Molded Pureed Foods in Community-Dwelling Older Adults in Singapore
Source: Nutrients. 2026 Oct 18;18(20):3413. doi: 10.3390/nu18203413 (PMC13708448; doi:10.3390/nu18203413)
Supplement: Supplementary file 1 [file nutrients-18-03413-s001.zip › nutrients-4590306-supplementary.pdf]

Supplementary Materials

Food Preferences and Acceptability of Molded Pureed Foods in Community-Dwelling Older Adults in Singapore

Table S1

Table S1. Complete food and snack category recall counts by chewing/swallowing difficulty status

Group	Category	Total recall	No difficulty (n=44)	Difficulty (n=56)	P-value	FDR	Flag
Main meal	Chinese noodle dishes	80	37	43	0.454	0.686	Ok
	Chicken dishes	62	29	33	0.537	0.686	Ok
	Pork dishes	46	20	26	1.000	1.000	Ok
	Malay dishes	39	15	24	0.414	0.686	Ok
	Seafood dishes	38	19	19	0.408	0.686	Ok
	Other Singaporean hawker favourites	29	11	18	0.509	0.686	Ok
	Indian dishes	14	5	9	0.572	0.686	Ok
	Vegetable dishes	9	1	8	0.073	0.337	Low group count
	Egg dishes	6	5	1	0.084	0.337	Low group count
	Bread with spread / filling	5	0	5	0.065	0.337	Low group count
	Duck dishes	4	1	3	0.628	0.686	Low group count
	Fruits	2	0	2	0.502	0.686	Low group count
Snacks	Savoury snacks	83	38	45	0.593	0.932	Ok
	Sweet snacks	80	38	42	0.210	0.932	Ok
	Deep-fried snacks	48	21	27	1.000	1.000	Ok
	Buns with filling	30	14	16	0.827	1.000	Ok
	Bread with spreads / fillings	13	7	6	0.553	0.932	Ok
	Biscuits and crackers	6	2	4	0.692	0.952	Low group count
	Non-caffeinated beverages	6	1	5	0.225	0.932	Low group count
	Fruits	5	1	4	0.381	0.932	Low group count
	Indian snacks	4	3	1	0.317	0.932	Low group count

Group	Category	Total recall	No difficulty (n=44)	Difficulty (n=56)	P-value	FDR	Flag
	Caffeinated beverages	3	1	2	1.000	1.000	Low group count
	Malay snacks	3	2	1	0.581	0.932	Low group count

Values are the number of participants recalling at least one item in the category (no difficulty, n=44; difficulty, n=56). P-values are from Fisher's exact test; FDR is the Benjamini–Hochberg-adjusted p-value, calculated separately within the food-category family (n=12) and the snack-category family (n=11). The flag column reports data sufficiency for each category; categories with fewer than 10 total recalls, or fewer than 3 recalls in either subgroup, are flagged "low group count"; all remaining categories are flagged “Ok”. The 11 flagged categories (5 food, 6 snack) were omitted from Table 5 in the main text; the 12 categories flagged "ok" (7 food, 5 snack) correspond to those reported there. No category, flagged or unflagged, remained significant after FDR correction.
